# Influenza vaccination and the risk of COVID-19 infection and severe illness in older adults in the United States

**DOI:** 10.1038/s41598-021-90068-y

**Published:** 2021-05-26

**Authors:** Kelly Huang, Shu-Wen Lin, Wang-Huei Sheng, Chi-Chuan Wang

**Affiliations:** 1 School of Pharmacy, College of Medicine, National Taiwan University, Room 202, No. 33, Linsen S. Rd., Zhongzheng Dist., Taipei City, 100 Taiwan; 2 Graduate Institute of Clinical Pharmacy, College of Medicine, National Taiwan University, Taipei, Taiwan; 3 Department of Pharmacy, National Taiwan University Hospital, Taipei, Taiwan; 4 Department of Internal Medicine, National Taiwan University Hospital, Taipei, Taiwan

**Keywords:** Viral infection, Outcomes research

## Abstract

The coronavirus disease of 2019 (COVID-19) has caused a global pandemic and led to nearly three million deaths globally. As of April 2021, there are still many countries that do not have COVID-19 vaccines. Before the COVID-19 vaccines were developed, some evidence suggested that an influenza vaccine may stimulate nonspecific immune responses that reduce the risk of COVID-19 infection or the severity of COVID-19 illness after infection. This study evaluated the association between influenza vaccination and the risk of COVID-19 infection. We conducted a retrospective cross-sectional study with data from July 1, 2019, to June 30, 2020 with the Claims data from Symphony Health database. The study population was adults age 65 years old or older who received influenza vaccination between September 1 and December 31 of 2019. The main outcomes and measures were odds of COVID-19 infection and severe COVID-19 illness after January 15, 2020. We found the adjusted odds ratio (aOR) of COVID-19 infection risk between the influenza-vaccination group and no-influenza-vaccination group was 0.76 (95% confidence interval (CI), 0.75–0.77). Among COVID-19 patients, the aOR of developing severe COVID-19 illness was 0.72 (95% CI, 0.68–0.76) between the influenza-vaccination group and the no-influenza-vaccination group. When the influenza-vaccination group and the other-vaccination group were compared, the aOR of COVID-19 infection was 0.95 (95% CI, 0.93–0.97), and the aOR of developing a severe COVID-19 illness was 0.95 (95% CI, 0.80–1.13). The influenza vaccine may marginally protect people from COVID-19 infection.

## Introduction

The coronavirus disease of 2019 (COVID-19) has caused a global pandemic and led to nearly three million deaths globally^1^. As of April 2021, there are still many countries that do not have COVID-19 vaccines. Before the COVID-19 vaccines were developed, there was some evidence suggesting that an influenza vaccine may stimulate nonspecific immune responses that reduce the risk of COVID-19 infection or the severity of COVID-19 illness after infection^2–8^.

Early evidence suggests that COVID-19 patients with positive influenza A immunoglobulin M (IgM) had a lower risk of mortality and severe COVID-19 illness compared with those who showed a negative IgM status^2^. Furthermore, it has been reported that influenza vaccination is associated with a lower risk of COVID-19 infection^3–5^. Studies have also shown that influenza vaccination is negatively associated with COVID-19 mortality in older adults^6,7^; in Brazil, a patient registry showed that patients who recently received an influenza vaccination had a lower odds of severe illness and mortality from COVID-19^8^. Recent studies have also found that influenza vaccination is associated with moderate risk reduction in all-cause hospitalization and mortality among patients diagnosed with COVID-19 infection^9^, and a lower risk of 60-day mortality among patients who tested positive for COVID-19 in an emergency department^10^.

Older adults and patients with comorbidities are at greater risk of COVID-19 infection, and they are also more likely to develop severe illness after infection^11,12^. Given that the influenza vaccine is safe and currently available, it may be a quick and safe option to slow down the COVID-19 pandemic. Limited evidence exists about the association between influenza vaccination and the incidence of COVID-19 in elder population. While prior research suggests that influenza vaccination may reduce the risk of COVID-19 infection, healthy vaccine effect was not considered. Therefore, it is important to further evaluate the effect of influenza vaccination on risk of COVID-19 infection taking healthy vaccine effect into consideration. Given that older adults are especially vulnerable to COVID-19, the aim of the present study is to evaluate whether influenza vaccination (1) reduces the risk of COVID-19 infection and (2) reduces the severity of illness after being infected by COVID-19 in adults age 65 or older.

## Methods

This cross-sectional observational study was conducted using the Symphony Health dataset (PRA Health Sciences, Raleigh, NC, USA) from the COVID-19 Research Database^13^. The COVID-19 Research Database was established with Institutional Review Board approval and an exemption from patient consent because it included only data considered to be de-identified by the Health Insurance Portability and Accountability Act (HIPAA), HIPAA-limited data, or non-HIPAA-covered data, along with the strong governance measures in place to control access to all data. The Symphony Health dataset was derived from pharmacy and medical claims from several sources including Medicare, which covers about 280 million patients (almost all older adults), 1.8 million prescribers, and 16,000 health plans in the United States.

**Figure 1:**
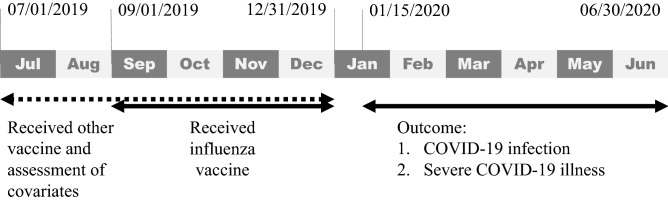
Timeline of covariate assessment, receipt of influenza or other vaccine, and outcomes.

We used data collected from July 1, 2019, to June 30, 2020, about older adults (age 65 years old or older). To identify incident COVID-19 cases, we excluded individuals who had received a COVID-19 diagnosis before January 15, 2020. We also excluded individuals who had received an influenza vaccination on or after January 1, 2020 for two reasons. First, it generally takes 14 days for the body to develop antibodies against influenza after vaccination, so we hypothesized that the effect of the influenza vaccination would begin 14 days after the vaccination^14^. Second, although our design is closer to a cross-sectional study, we used the 14-day window to strengthen the temporality between influenza vaccination and COVID-19 infection. All the exposure (that is, influenza vaccination) occurred before the outcome (that is, COVID-19 infection). Figure 1 shows the timeline of covariate assessment, receipt of influenza or other vaccine, and outcomes.

We classified individuals into an influenza-vaccination group and a no-influenza-vaccination group on the basis of their influenza vaccination status between September 1, 2019, and December 31, 2019. The differences among vaccines were considered as covariates, including trivalent or quadrivalent, live attenuated or inactivated, and with or without an adjuvant. The intranasal influenza vaccine was not recommended to older adults, and we did not find any intranasal influenza vaccine users in our study sample. The National Drug Codes for the influenza vaccines and other vaccines were from the Centers for Disease Control (CDC)^15^. Two outcomes were identified on and after January 15, 2020 in this study: incidence of COVID-19 infection and incidence of severe illness because of COVID-19 infection. These two outcomes follow the U.S. Food and Drug Administration’s definition (Table S1)^16^.

The covariates included in this study were as follows: age 75 or older, gender, vaccinated against a disease other than influenza between July 1 and December 31, whether the influenza vaccine contained an adjuvant, and comorbidities that may increase the risk of COVID-19. The four types of vaccines that are recommended by the CDC and commonly administered to older adults were adjusted in Analysis 1 and served as comparators in Analysis 2 (that is, herpes zoster, pneumococcal pneumonia, tetanus, and hepatitis A). The influenza vaccines included in this study were mainly FLUZONE High-Dose (Sanofi Pasteur Inc.) and FLUAD (Seqirus USA Inc.), which accounted for 56% and 29% of the study sample, respectively. These were the two vaccines that were recommended for older adults by the CDC. The use of other influenza vaccines was less than 5%. Both vaccines were trivalent in the study period; FLUZONE High-Dose is a vaccine without an adjuvant; FLAUD contains an adjuvant. Thus, we did not control the vaccine valency; instead, we adjusted the appearance of the adjuvant in multivariate analyses because a vaccine adjuvant may reduce COVID-19 severity^17^. Comorbidities were selected according to the CDC’s warning about at-risk populations, including asthma, chronic kidney disease with dialysis, chronic lung disease, diabetes mellitus, hemoglobin disorders, immunocompromised, liver disease, serious heart conditions, and severe obesity (Table S1)^18^.

Two analyses were performed in this study. We first compared the odds of contracting COVID-19 between individuals who received an influenza vaccination versus those who did not (Analysis 1). To clarify the healthy vaccine effect, we repeated the aforementioned analyses by comparing individuals who received an influenza vaccination to those who receive a vaccination other than for influenza (Analysis 2). Vaccines that are recommended for older adults were selected as the comparator (herpes zoster, pneumococcal pneumonia, tetanus, and hepatitis A).

The risk of COVID-19 infection and severe COVID-19 illness were evaluated with univariate and multivariate logistic regressions. To compare the influenza-vaccination group with the no-influenza-vaccination group, we divided the study cohort into 2000 subcohorts because computing efficiency limited the processing of such a large amount of data at once. We calculated the odds ratio in each subcohort and used a meta-analysis approach to get the pooled results. The final result was pooled using a random-effects model. Data were managed using the Snowflake® data warehouse (Snowflake Inc., San Mateo, CA, USA), and the analyses were performed using SAS® version 9.4 (SAS Institute Inc., Cary, NC, USA).

## Results

**Figure 2:**
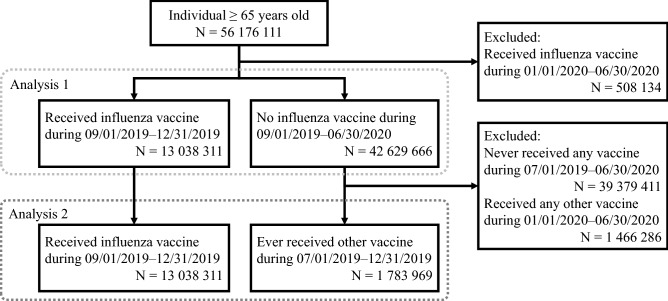
Flow chart of patient selection and comparisons.

The Symphony Health dataset included records from 56 million older adults (Fig. 2). About 13 million older adults received the influenza vaccination between September 1 and December 31 of 2019. More than 42 million older adults who did not receive an influenza vaccination were selected as a control group for the comparison in Analysis 1. In the same period, 4.7 million older adults received other vaccines (herpes zoster, pneumococcal pneumonia, tetanus, and hepatitis A), and nearly 1.8 million of them had not received an influenza vaccine. These 1.8 million patients were selected as a control group in Analysis 2.

The characteristics of the study cohorts are in Table 1. Individuals who had received either an influenza vaccination or other non-influenza vaccination tended to have a lower comorbidity burden compared to individuals who did not receive an influenza vaccination. Among individuals who did not receive an influenza vaccination, 1.2% of them had a COVID-19 infection and 0.02% of them developed severe COVID-19 illness. Those who received an influenza vaccination had a COVID-19 infection rate of 0.9% and a severe case rate of 0.01%. Those who received a vaccination for something other than influenza had a COVID-19 infection rate of 0.98% and a severe COVID-19 illness case rate of 0.01%. Those descriptive statistics show that older adults who received an influenza vaccination had the lowest rate of COVID-19 infection and the lowest severe COVID-19 illness case rate.

**Table 1:** Characteristics of the three study cohorts: no influenza vaccination, influenza vaccination, and vaccination other than influenza.

Characteristics	No influenza vaccination	Percent	Influenza vaccination	Percent	Vaccination other than influenza	Percent
**N**	42,629,666		13,038,311		1,783,969	
**Age 75 and older**	17,909,748	42	5,568,818	43	671,574	38
**Male**	19,042,518	45	5,636,245	43	739,562	41
**Risk factors**						
Asthma	91,734	0.22	31,753	0.24	5052	0.28
Chronic kidney disease with dialysis	43,809	0.10	1787	0.014	888	0.050
Chronic lung disease	1,520,026	3.6	365,723	2.8	50,698	2.8
Diabetes mellitus	4,620,287	11	1,165,787	8.9	175,659	9.8
Hemoglobin disorder	17,624	0.041	4285	0.033	776	0.043
Immunocompromised	214,861	0.50	59,518	0.46	9384	0.53
Liver disease	158,425	0.37	32,516	0.25	5973	0.33
Serious heart condition	3,528,349	8.3	891,346	6.8	122,178	6.8
Severe obesity	1,511,601	3.5	423,755	3.3	67,534	3.8
**Other vaccination**^**1**^	34,669,841	81	8,004,874	61	Not applicable	
**Vaccine with adjuvant**	Not applicable		3,740,784	29	Not applicable	
**Outcome**						
COVID-19 infection	532,550	1.2	117,467	0.90	17,472	0.98
Severe COVID-19 illness	8540	0.020	1302	0.010	223	0.013

^1^Other vaccines: herpes zoster, pneumococcal pneumonia, tetanus, and hepatitis A.

**Figure 3:**
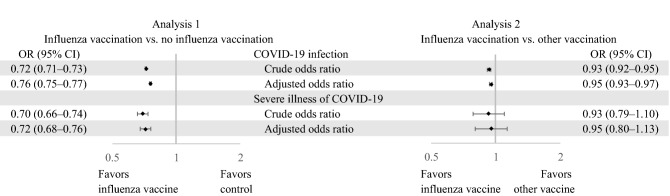
Effectiveness of influenza vaccine for protection against COVID-19 infection and severe COVID-19 illness. OR, odds ratio; CI, confidence interval.

Without adjusting for any risk factors, the odds of getting COVID-19 infection for the influenza-vaccination group was 0.72 times that of the no-influenza-vaccination group, with a 95% confidence interval (95% CI) of 0.71–0.73 (Fig. 3). The adjusted odds ratio (aOR) from the pooled analysis was 0.76 (95% CI 0.75–0.77). The distributions of the aORs and their lower and upper limits from the 2000 subcohorts are shown in Fig. S1. Among COVID-19 patients, the crude odds ratio (OR) of developing severe illness was 0.70 (95% CI 0.66–0.74) in patients who had received an influenza vaccination compared to patients who did not receive an influenza vaccination, and the aOR was 0.72 (95% CI 0.68–0.76).

In Analysis 2, the crude OR of COVID-19 infection between the influenza-vaccination group and other-vaccination group was 0.93 (95% CI 0.92–0.95), and the multivariate aOR was 0.95 (95% CI 0.93–0.97). Among COVID-19 patients, the unadjusted OR of severe COVID-19 illness was 0.93 (95% CI 0.79–1.10) when the influenza-vaccination group was compared with the other-vaccination group, and the aOR was 0.95 (95% CI 0.80–1.13).

## Discussion

In this study, older adults who had received an influenza vaccination were associated with a 24% reduction in the odds of getting a COVID-19 infection and a 28% reduction in the odds of developing a severe COVID-19 illness, compared to older adults who had not received influenza vaccination. When we compared individuals who had received an influenza vaccination to those who received a non-influenza vaccination, the protective effect against COVID-19 infection was reduced from 24% to 5% but remained significant. Receiving an influenza vaccination did not reduce the odds of developing severe illness in patients with COVID-19 infection when compared to receiving a non-influenza vaccination.

Our results suggest that an influenza vaccination seems to have a protective effect against COVID-19 infection, which implies that an influenza vaccination may trigger nonspecific immune responses that help protect against COVID-19 infection. This finding was also consistent with prior evidence that suggested that an influenza vaccination may reduce the risk of a COVID-19 infection or severe COVID-19 illness^3–8^. There is also a hypothesis that immune responses in the influenza vaccine may induce bystander immunity against SARS-CoV-2^17^.

Although receiving an influenza vaccination was associated with a significant reduction in the risk of COVID-19 infection compared to not receiving an influenza vaccination, this effect may come from the healthy vaccine effect because people who were vaccinated may be healthier in general than those who were not vaccinated^19^. In addition, individuals who were vaccinated often have other health behaviors that may prevent the transmission of COVID-19 or reduce the severity of COVID-19 illness. The healthy vaccine effect may be reflected by the result that only a marginal effect was found when we compared the risk of COVID-19 infection between those who received an influenza vaccine and those who received a vaccine against something other than influenza; no difference in the risk of severe COVID-19 illness was observed between the influenza-vaccination group and the no-influenza-vaccination group. On the other hand, the marginal protective effects of influenza vaccination may be underestimated in this analysis as vaccination may increase individuals’ immunity against diseases in general^20^.

Despite the positive findings, a few studies have reported a null association between influenza vaccination and risk of COVID-19 infection^21–23^. These studies defined COVID-19 infection by laboratory-confirmed cases instead of assessing clinical outcomes after infection; two of them evaluated the effects among healthcare workers, and none of them reported the results for individuals aged ≥ 65. Because healthcare workers and younger adults generally have better immunity, influenza vaccination may provide insignificant benefit effect against COVID-19 infection in these populations. Among individuals aged ≥ 65, influenza vaccination is associated with a slightly lower probability of COVID-19 infection as well as lower odds of hospitalization and death^4,24^.

### Study limitation

In this study, we selected four other vaccines that are recommended by the CDC to the older adults as comparators to avoid the healthy vaccine effect. However, vaccines against diseases other than influenza are only administered once in a lifetime or at an interval of many years in between vaccinations. In the present study, only older adults who had received a vaccination after July 2019 could be traced. Therefore, a potential misclassification of this covariate may exist. The results of the comparison between the influenza-vaccination group and the non-influenza-vaccination group should be conservative because individuals were required to have received at least one non-influenza vaccination to be included in the comparison group.

Although the ethnicity and geographic location might affect the incidence of COVID-19 infection, we were unable to adjust for these two variables. In the Symphony Health dataset, 35% of older adults had no ethnic information, and only the first two digits of their zip codes were provided.

Finally, we were unable to implement self-control or case-crossover designs to avoid the healthy vaccine effect because our data only covered one year^19^. However, one important assumption of the case-crossover design is that the outcome event can occur “bi-directionally" (that is, outcomes can occur before and after the exposure), and the event could be reversible^25^. Given that COVID-19 has been newly detected this year, it violates the bi-directional assumption. In short, it is impossible to identify an outcome event (that is, a COVID-19 case) before the exposure (that is, an influenza vaccination) because COVID-19 was not identified before 2020.

## Conclusion

The influenza vaccine may only marginally protect people from COVID-19 infection. However, it remains important to receive an influenza vaccination to reduce the risk of a co-infection of influenza and COVID-19. Because influenza and COVID-19 present with similar symptomatology and occupy the same medical resources, the influenza vaccine is crucial in reducing the number of severe influenza patients in order to free up resources that may be necessary to handle another wave of COVID-19 patients.

## Supplementary Information

Supplementary Information.

Supplementary Figure S1.
