# Supplementary material for: Influenza vaccination and the risk of COVID-19 infection and severe illness in older adults in the United States
Source: Sci Rep. 2021 May 26;11:11025. doi: 10.1038/s41598-021-90068-y (PMC8155195; doi:10.1038/s41598-021-90068-y)
Supplement: Supplementary file 1 — Supplementary Information. [file 41598_2021_90068_MOESM1_ESM.pdf]

Influenza Vaccination and the Risk of COVID-19 Infection and Severe Illness in Older Adults in the United States

Kelly Huang, Shu-Wen Lin, Wang-Huei Sheng, Chi-Chuan Wang

Supplementary Information

Table S1. Definition and coding of diseases

Item	Definition	Coding system	Code
COVID-19 infection	Dx: Coronavirus infection	ICD-10	B34.2, B97.29
	Dx: SARS-associated coronavirus	ICD-10	B97.21, J12.81
	Dx: COVID-19	ICD-10	U07.1, U07.2
	Dx: Exposure to other viral communicable diseases	ICD-10	Z20.828
Severe COVID-19 illness	Pcs: Mechanical ventilation	CPT	31500, 1015098, 1022227
Risk factors			
Asthma	Dx: Moderate to severe asthma	ICD-10	J45.4, J45.5
CKD with dialysis	Pcs: Dialysis	CPT	90935, 90937
Chronic lung disease	Dx: COPD	ICD-10	J44.9
Diabetes	Dx: Diabetes	ICD-10	E08-E13
Hemoglobin disorder	Dx: Thalassemia, sickle cell disease	ICD-10	D56, D57
Immunocompromised	Dx: Human immunodeficiency virus disease	ICD-10	B20
	Pcs: Cluster of differentiation 4 receptors test	CPT	86359, 86360, 86361
	Dx: Immunodeficiency	ICD-10	D83
	Dx: Transplant	ICD-10	Z94
Liver disease	Dx: Cirrhosis, chronic hepatic failure	ICD-10	K72-K74
Serious heart condition	Dx: Heart failure, chronic ischemic heart disease, pulmonary circulation disease	ICD-10	I50, I25-I28
Severe obesity	Dx: Obesity	ICD-10	E66

Dx, diagnosis; Pcs, procedure; ICD-10, International Classification of Disease, 10th revision; CPT, Current Procedural Terminology; COVID-19, coronavirus disease 2019; SARS, severe acute respiratory syndrome; CKD, chronic kidney disease; COPD, chronic obstructive pulmonary disease.

Figure S1. The distribution of odds ratio in 2000 subcohorts

(Left: odds ratio; middle: lower limit of 95% confidence interval; right: upper limit of 95% confidence interval)
