# Supplementary figures and images for: Influenza vaccination and the risk of COVID-19 infection and severe illness in older adults in the United States
Source: Sci Rep. 2021 May 26;11:11025. doi: 10.1038/s41598-021-90068-y (PMC8155195; doi:10.1038/s41598-021-90068-y)

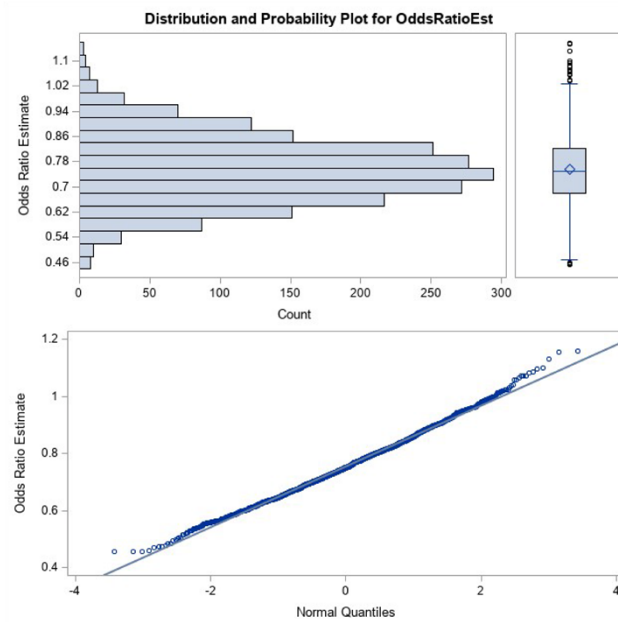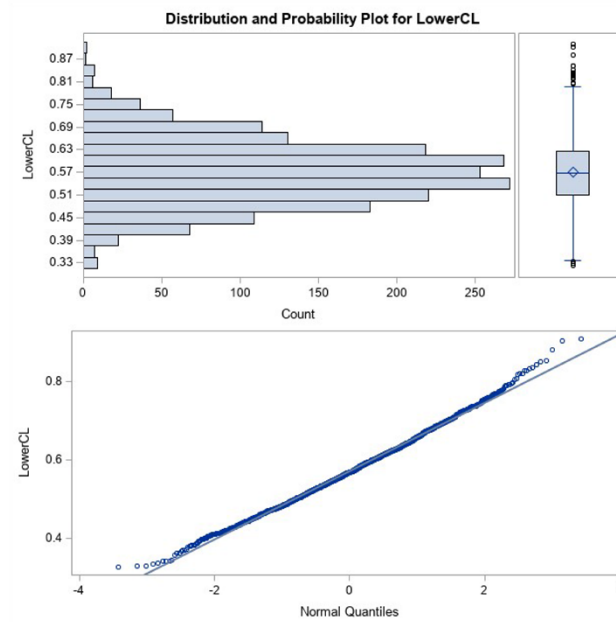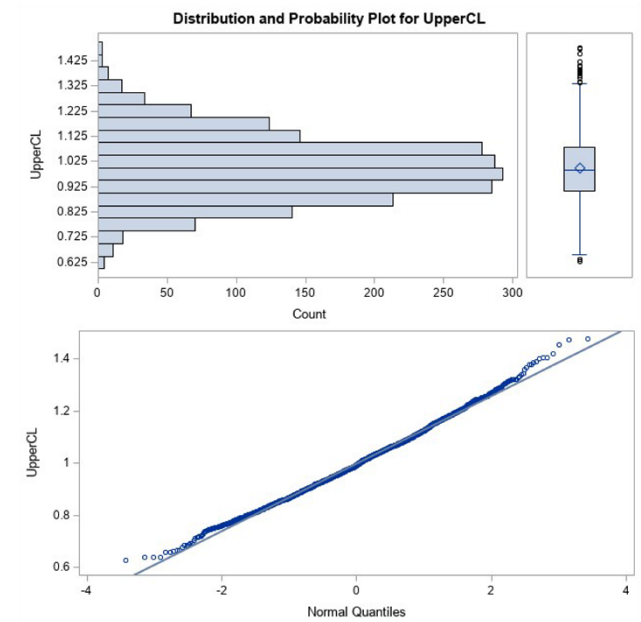

Supplement: Supplementary file 2 — Supplementary Figure S1. [file 41598_2021_90068_MOESM2_ESM.pdf]
